# Les cancers cutanés à Madagascar: où en sommes-nous?

**DOI:** 10.11604/pamj.2019.34.167.19269

**Published:** 2019-11-29

**Authors:** Andrianarison Malalaniaina, Tika Lovasoa, Ranaivo Irina Mamisoa, Razakanaivo Malala, Ramarozatovo Lala Soavina, Rafaramino Florine, Rapelanoro Rabenja Fahafahantsoa

**Affiliations:** 1 Service Dermatologie, Centre Hospitalo-Universitaire Joseph Raseta Befelatanana, Antananarivo, Madagascar; 2 Service Oncologie, Centre Hospitalo-Universitaire Joseph Ravoahangy Andrianavalona, Antananarivo, Madagascar; 3 Service Dermatologie, Centre Hospitalo-Universitaire Morafeno Tamatave, Madagascar; 4 Service de Médecine Interne et de Dermatologie, Centre Hospitalo-Unversitaire Joseph Raseta Befelatanana, Antananarivo, Madagascar

**Keywords:** Cancers cutanés, carcinomes, épidémiologie, mélanome, Madagascar, Skin cancers, carcinomas, epidemiology, melanoma, Madagascar

## Abstract

**Introduction:**

Les cancers cutanés sont des tumeurs kératinisantes malignes, d’origines épidermique, mélanique ou annexielle. Ces cancers sont encore peu connus, pourtant existent bien à Madagascar, un pays abritant un peuple à phototype diversifié sous un fort degré d’ensoleillement. Notre étude vise à déterminer le profil épidémio-clinique des cancers cutanés à Antananarivo.

**Méthodes:**

Il s’agit d’une étude rétrospective, descriptive des cancers cutanés sur une durée de sept ans au Service d’Oncologie du CHU Joseph Ravoahangy Andrianavalona, Antananarivo. Elle inclut les patients présentant cliniquement des lésions cutanées d’allure tumorale, confirmées ensuite par un examen histologique.

**Résultats:**

Nous avions colligé 47 cas de cancers cutanés à prédominance masculine (sex ratio 1,13). L’âge moyen était de 49,3 ans. Les agriculteurs étaient les plus touchés. Les lésions touchaient préférentiellement la tête, le cou (44%) et les membres inférieurs (42%). Les cancers cutanés étaient découverts au stade localisé dans 61,7% des cas. Le carcinome épidermoïde constituait le premier cancer cutané des Malgaches (37%), suivi par le mélanome (21%) et le carcinome basocellulaire (11%). La prise en charge était essentiellement chirurgicale (74,19%).

**Conclusion:**

Le cancer épidermoïde est le premier cancer Malgache, suivi du mélanome et du carcinome basocellulaire. Les cancers cutanés touchent surtout la population jeune nécessitant une prise en charge bien cadrée.

## Abstract (en)

**Introduction:**

Skin cancers are malignant keratinizing tumors of epidermal, melaninic or adnexal origin. These cancers are still little known. However they affect people living in Madagascar where the amount of sunshine remains very high with an impact that varies depending on the skin type. The purpose of our study was to determine the epidemio-clinical profile of skin cancers in Antananarivo.

**Methods:**

We conducted a retrospective descriptive study of skin cancers in the Department of Oncology of the Joseph Ravoahangy Andrianavalona Hospital, Antananarivo over a period of seven years. It included patients with tumoral skin lesions confirmed by histological examination.

**Results:**

We collected data from 47 cases of skin cancers (male predominance; sex ratio 1.13). The average age of patients was 49.3 years. Farmers were the most affected. The lesions mainly occurred in the head, neck (44%) and lower limbs (42%). Localized skin cancers were detected in 61.7% of cases. Squamous cell carcinoma was the most common skin cancer in Madagascar (37%), followed by the melanoma (21%) and the basal cell carcinoma (11%). Treatment was mainly based on surgery (74.19%).

**Conclusion:**

Squamous cell carcinoma is the most common cancer in Madagascar, followed by melanoma and basal cell carcinoma. Skin cancers affect mainly the young population and require appropriate management.

## Introduction

Les cancers cutanés sont des tumeurs malignes qui se développent au dépend de l’un des constituants de la peau, ils peuvent être d’origine épidermique, mélanique, ou annexielle. D’après Glass, Gray, Alam et Lomas, l’incidence des carcinomes épidermoïdes cutanés semble croître de 50 à 200% durant ces 30 dernières années [1-4]. Avril *et al.* avaient constaté que le mélanome cutané était l’un des cancers dont l’incidence augmentait le plus au cours des dernières décennies, en particulier dans les pays occidentaux industrialisés [5]. De même chez les hispaniques américains, cette incidence a augmenté de 11 % entre 1992 et 2002 [6]. Le carcinome épidermoïde et le mélanome ont un fort pouvoir métastatique [7-9], tandis que le carcinome basocellulaire est un cancer très délabrant et handicapant pouvant mettre en jeu le pronostic fonctionnnel et esthétique du patient. Depuis ces cinq dernières années, avec l’avènement de l’immunothérapie et la thérapie ciblée, la prise en charge des cancers cutanés a connu de grand progrès avec une efficacité concrète marqué par un prolongement de la survie globale dans les pays occidentaux. La peau malgache n’est pas à l’abri de ces cancers. En effet, le haut degré d’ensoleillement à Madagascar couplé par une exposition solaire chronique des agriculteurs et des travailleurs peu ou non protégés exposent aux Malgaches un grand risque. Cette pathologie est encore très mal connue à Madagascar car peu d’études sont en cours. Ainsi, l’objectif principal de notre étude est de décrire le profil épidémio-clinique de ces cancers cutanés vus dans le Service d’Oncologie-Radiothérapie de l’hôpital Joseph Ravoahangy Andrianavalona (CHU-JRA) de 2008 à 2015. Ceci afin d’une part de déterminer leurs particularités cliniques, paracliniques et thérapeutiques et d’autre part de sensibiliser tous les acteurs médicaux pour une meilleure prise en charge multidisciplinaire.

## Méthodes

Nous avons mené une étude rétrospective descriptive allant du 1er janvier 2008 au 31 décembre 2015, soit une durée de 8 ans dans le Service d’Oncologie-Radiothérapie du CHU-JRA. Nous avons inclus dans cette étude tous les patients ayant des preuves histologiques de leur cancer cutané primitif. Les paramètres étudiés étaient l’incidence annuelle, l’âge, le genre, le secteur d’activité, la localisation initiale de la tumeur, la taille lésionnelle, le stade de la maladie, le type histologique et enfin les armes thérapeutiques disponibles et réalisables dans notre pays.

## Résultats

Durant la période d’étude de 2008 à 2015, 9391 patients étaient vus au sein du Service d’Oncologie-Radiothérapie du Centre Hospitalier Joseph Ravoahangy Andrianavalona d’Antananarivo toutes consultations confondues. Nous avions colligés 47 cas de cancers cutanés soit 0,5% de tous les cancers recensés dans le service. L’incidence des cancers cutanés en 2008 était de 6,38% et en 2015 elle était de 25,53; soit une augmentation de quatre fois plus en sept ans (Tableau 1). L’âge des patients atteints de cancers cutanés variait de 25 à 73 ans, avec une moyenne de 49,3 ans. La tranche d’âge de 50 à 59 ans était la plus touchée, représentant 31,91 % des cas. Les agriculteurs étaient les plus touchés représentant 42,55 % de la population atteinte. Les cancers cutanés se localisaient préférentiellement au niveau des régions de la tête et du cou (44%) dont 12 cas (25,53%) au niveau du cuir chevelu et 9 cas (19,15%) au niveau de la face. Les membres inférieurs étaient la deuxième localisation (42%) prédominée par la localisation au niveau des pieds chez 11 cas (23,41%). L’atteinte du tronc était retrouvée chez 4 patients (8,51%). Les lésions mesuraient en moyenne 7,2cm dont 53,19% étaient comprises entre 5-10cm. Cliniquement, les lésions se présentaient sous formes bourgeonnantes chez 29 cas (61%) et ulcéreuses chez 12 patients (26%). Les deux formes étaient retrouvées chez 6 patients (13%). Le carcinome épithélial prédominait le type histologique des cancers cutanés dont le carcinome épidermoïde à 37 % et le carcinome basocellulaire à 11 %. Le mélanome constituait le deuxième cancer cutané avec un taux de 21 % des cas de cancer, dont 92% étaient des mélanomes plantaires. Les autres cancers cutanés étaient le liposarcome (N = 3; 6%), sarcome de Kaposi (N = 3; 6%) et les autres types de cancers.(Tableau 2). Par ailleurs, 19 patients étaient directement référés au centre d’oncologie dès la première consultation en médecine générale, dont 7 patients (15%) vus par des dermatologues. Néanmoins 2 patients consultaient 5 à 8 fois en médecine générale avant d’être vus en Oncologie. Parmi les 47 patients, 66% avaient bénéficié d’un traitement (soit 31 patients).34% n’ont pas pu être traités, du fait de la localisation tumorale difficilement accessible à la chirurgie et surtout de la perte de vue précoce des patients. La chirurgie constituait le principal traitement des cancers cutanés dans 74,19% des cas. La radiothérapie n’était indiquée que dans 9,68% des cas.

**Tableau 1:** Nombre annuelle des cancers cutanés de 2008 à 2015

Années	2008	2009	2010	2011	2012	2013	2014	2015
n	3	4	7	8	5	4	4	12
%	6,38	8,51	14,89	17,02	10,64	8,51	8,51	25,53

**Tableau 2:** Répartition des types histologiques des cancers cutanés au cours de l’étude

Types histologiques	Effectifs n =	Pourcentage (%)
Carcinome épidermoïde	17	37
Mélanome	10	21
Carcinome basocellulaire	9	19
Autres	5	11
Liposarcome	3	6
Sarcome de Kaposi	3	6

## Discussion

**Figure 1:**
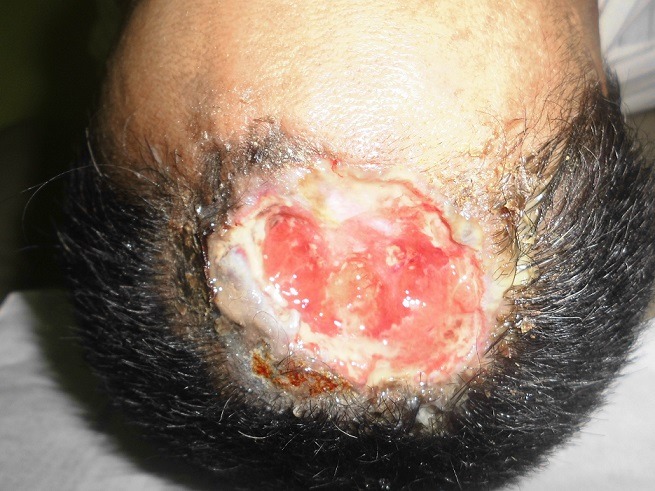
Carcinome épidermoïde de cuir chevelu

**Figure 2:**
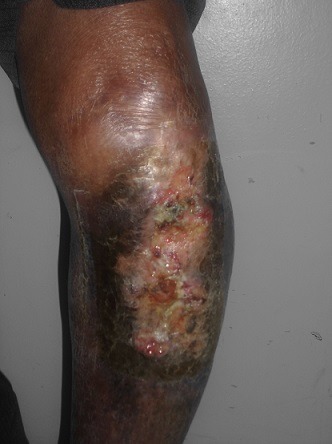
Carcinome basocellulaire de la jambe gauche

D’après cette étude, la prévalence des cancers cutanés à Madagascar est faible. Cette prévalence est pourtant élevée chez d’autres pays comme en Afrique, en Burkina Faso, Ouedraogo *et al.* mettaient en évidence 30 cas en 2013 sur une période de 8 mois [10]. En Tunisie, Mseddi *et al.* recensaient 1476 cas en 24 ans, de 1979 à 2002, avec une incidence moyenne à 61,5 cas par an [11]. Cette fréquence élevée était également observée aux Antilles françaises où 164 cas de cancers cutanés étaient diagnostiqués en 2007, en espace de 3 mois [12]. Cette différence peut être expliquée par le nombre limité des centres de diagnostic et thérapeutique dans notre pays donnant des résultats sous-estimés. Mais parallèlement aux autres pays, l’incidence des cancers cutanés à Madagascar tend à augmenter, comme chez les hispaniques américains où l’incidence avait augmenté de 11% entre 1992 et 2002 [6]. Cette augmentation de l’incidence serait due au changement d’habitudes sur l’exposition solaire et à une meilleure collaboration entre médecins spécialisés et généralistes améliorant la référence des patients. La population de cette étude est jeune avec un âge moyen de 46 ans. Cette jeunesse se rencontre aussi en Afrique où l’âge moyen de diagnostic est de 48,5 ans [10, 11, 13]. La prédominance masculine était retrouvée dans de nombreuses études notamment les études africaines [12-14] et subsahariennes [15-17]. Les hommes agriculteurs seraient plus exposés, au rayonnement ultraviolet, aux frottements, aux ulcères chroniques et aux cicatrices. Selon Ouedraogo *et al*. il y a une nette prédominance des cancers cutanés chez les agriculteurs et les ménagères dans 73,3% des cas [10]. Glanz *et al.* avaient également rapporté que les travailleurs au grand air comme les agriculteurs sont les sujets les plus exposés professionnellement [18, 19]. La localisation céphalique prédominait dans la littérature (Figure 1) [10, 12, 20]. Les régions de la tête et du cou sont des zones photoexposées et les membres inférieurs sont des zones de traumatismes d’où la fréquence du cancer à ces endroits. Les carcinomes épithéliaux constituaient le premier cancer de notre série, suivis par le mélanome. Le carcinome épidermoïde était de loin le plus dominant. Cette classification se rapproche à celle de la littérature sauf une prédominance du carcinome basocellulaire sur les peaux à phototype plus claires (Figure 2). Par ailleurs, l’exposition chronique et cumulée aux ultra-violets serait le principal facteur de risque de Carcinome épidermoïde. Tandis qu’une exposition solaire intense, intermittente entrainerai un carcinome basocellulaire [21]. Madagascar, étant une île avec ses 1500 kilomètres de côtes, expose à sa population une exposition solaire très tôt et très forte d’où la fréquence particulière de cette pathologie chez les Malgaches. D’autant plus que l’utilisation de la photo-protection n’est pas encore dans les habitudes des Malgaches de par son prix et son accessibilité. La chirurgie constitue le traitement de référence des cancers cutanés localisés [10, 14, 22, 23]. L’indication de la radiothérapie est considérable, de 33,33 à 66,67% selon le type histologique [24, 25] et le stade de la maladie. Malheureusement, dans notre série, le nombre perte de vue dès la première consultation est élevé. La chirurgie première était incomplète et sans reprise dans plus de la moitié des cas majorant le pronostic sombre de la maladie.

## Conclusion

Les cancers cutanés étaient peu étudiés et sous-diagnostiqués à Madagascar. Le phototype foncé des Malgaches n’est pas à l’abri du cancer cutané surtout en pays à fort ensoleillement. Les cancers cutanés étaient peu étudiés et sous-diagnostiqués à Madagascar. Cette étude a démontré que les Malgaches sont un bel exemple d’amalgame de couleur et de phototype, mais que ce mélange constitue un grand facteur de risque de cancer cutané. En effet, tous les types de cancers retrouvés essentiellement autant sur la peau caucasienne, qu’africaine sont retrouvés chez les Malgaches.

### Etat des connaissances actuelles sur le sujet

- L’incidence des carcinomes épidermoïdes cutanés semble croître de 50 à 200% durant ces 30 dernières années;
- Le mélanome cutané était l’un des cancers dont l’incidence augmentait le plus au cours des dernières décennies, en particulier dans les pays occidentaux industrialisés;
- Depuis ces cinq dernières années, avec l’avènement de l’immunothérapie et la thérapie ciblée, la prise en charge des cancers cutanés a connu de grands progrès avec une efficacité concrète marquée par un prolongement de la survie globale dans les pays occidentaux.

### Contribution de notre étude à la connaissance

- Cette étude situe le profil épidémio-clinique des cancers cutanés Malgaches par rapport aux autres pays d’Afrique;
- Elle réitère la gravité du mélanome acral chez la population à phototype foncée;
- L’étude confirme l’importance des carcinomes épithéliaux dans les pays à fort degré d’ensoleillement.

## Conflits d’intérêts

Les auteurs ne déclarent aucun conflit d'intérêts.
